# Supplementary material for: A Pattern of Care Report on the Management of Patients with Squamous Cell Carcinoma of the Anus—A Study by the Italian Association of Radiotherapy and Clinical Oncology (AIRO) Gastrointestinal Tumors Study Group
Source: Medicina (Kaunas). 2021 Dec 9;57(12):1342. doi: 10.3390/medicina57121342 (PMC8707820; doi:10.3390/medicina57121342)
Supplement: Supplementary file 1 [file medicina-57-01342-s001.zip › medicina-1437999-supplementary.pdf]

AIRO – Study group for Gastrointestinal malignancies

Italian investigational survey on the pattern of practice in the multimodal treatment of squamous cell carcinoma of the anus

Anal squamous cell carcinoma is a rare neoplasm, with a growing incidence in Western countries. Evidence from several phase III randomized trials have established definitive radio-chemotherapy as the standard of care. However, some issues related to diagnosis, radiotherapy options in terms of doses, volumes and techniques, as well as systemic therapy, remain controversial. Our Italian survey, proposed by the AIRO study group for Gastrointestinal malignancies, aims to investigate the most common approaches in the management of anal canal cancer patients, in order to find out potential 'gray zones' liable for study initiatives, educational programs, and consensus documents. Your answer to the survey will help us to selectively target research and treatment optimization in this clinical setting. Therefore, we thank you for your contribution

General profile of the respondent

1) Radiation Oncology Center:

a) Public
b) Private
c) Private in agreement with Public
d) Academic
e) Clinical Academic
f) Research Institute

2) In which Italian Region do you work?
Free text

3) How many years have you been treating squamous anal cancer?

a) < 5 years
b) 5-10 years
c) 11-15 years
d) > 15 years

4) How many patients diagnosed with anal squamous carcinoma are treated annually with radiotherapy in your Radiation Oncology Center?

a) < 10 patients
b) 10-20 patients
c) 21-30 patients
d) > 30 patients

5) Is there a multidisciplinary tumor board for lower gastrointestinal cancers in your center?

a) yes
b) no

Specific questions

6) Which examinations do you use in the initial diagnosis and staging of anal canal cancer (multiple answers allowed)?

a) anoscopy
b) colonoscopy
c) gynecological examination + colposcopy
d) thorax, abdomen and pelvis contrast-enhanced CT
e) contrast-enhanced high resolution pelvic MRI including inguinal region

f) ^{18}FDG-PET CT
g) endo-anal ultrasound

7) How do you consider pelvic MRI as staging exam for squamous anal cancer?

a) a mandatory examination
b) an optional but useful examination
c) a second level examination (in case of clinical doubt)
d) an unnecessary examination

8) How do you consider ^{18}FDG-PET for tumor and nodal staging of squamous carcinoma of the anus?

a) a mandatory examination
b) an optional but useful examination
c) a second level examination (in case of clinical doubt)
d) an unnecessary examination

9) In case of suspected involvement of one (or more) inguinal lymph nodes (diameter > 1 cm), do you suggest a biopsy/ fine needle aspiration?

a) always
b) only in case of a clinically palpable node, suspicious at CT imaging (diameter> 1 cm), with a positive finding at ^{18}FDG-PET
c) in case of a clinically palpable node, suspicious at CT imaging (diameter> 1 cm), with a suspicious ^{18}FDG-PET uptake
d) in case of a clinically palpable node, suspicious at CT imaging (diameter> 1 cm), without an ^{18}FDG-PET uptake
e) never

10) Do you usually request HIV screening on blood and/or saliva?

a) always
b) sometimes
c) only in case of risky behaviors
d) never

11) In your centre, when is HPV screening carried out on biopsy using immunohistochemical examination?

a) always
b) sometimes
c) only in young patients
d) according to investigational protocols
e) never (reason why:....)

12) Which role does the multidisciplinary tumor board play in the staging and treatment approach proposed to patients with squamous cell carcinoma of the anus?

a) standard management for all patients
b) employed only in selected cases
c) not applicable to my routine clinical practice

13) Which imaging modality do you usually prefer, in addition to simulation CT, when defining treatment volumes? (multiple answers allowed)

a) None, I use simulation CT only
b) Contrast-enhanced pelvic CT
c) Contrast-enhanced pelvic MRI
d) ^{18}FDG-PET CT

14) Which radiotherapy technique do you prefer for patients with anal cancer? (multiple answers allowed)

dd) 3DCRT
ee) IMRT
ff) VMAT
gg) Tomotherapy
hh) MRgRT

15) Which external beam radiotherapy (EBRT) technique do you prefer to deliver a boost dose? (multiple answers allowed)

ii) EBRT - sequential boost with photons
jj) EBRT - sequential boost with electrons
kk) EBRT - concomitant or simultaneous integrated boost (SIB)
ll) Endocavitary brachytherapy
mm) Interstitial brachytherapy
nn) Others (specify:…)

16) Which is your typical the prescription dose for elective nodal volumes?
[total dose to positive nodes= …..Gy/[dose/fraction]……Gy/[number of fractions…….]

Comments:….

17) Which is the prescription dose for macroscopically involved nodes?

Node < 3 cm - [total dose to positive nodes= …..Gy/[dose/fraction]……Gy/[number of fractions…….]

Comments:….

Node ≥ 3 cm [total dose to positive nodes= …..Gy/[dose/fraction]……Gy/[number of fractions…….]

Comments:….

18) Which is the prescription dose to primary tumor for stage cT1-T2 disease?

[total dose to primary tumor= …..Gy/[dose/fraction]……Gy/[number of fractions…….]

Comments:….

19) Which is the prescription dose to primary tumor for stage cT3-T4 disease?

[total dose to primary tumor= …..Gy/[dose/fraction]……Gy/[number of fractions…….]

Comments:….

20) In case of unresected cT1N0 anal margin tumor or in case of local excision of anal margin tumor (pT1N0) associated to risk factors at histological examination, what treatment would you consider?

oo) Definitive radiotherapy with curative dose
pp) Radio-chemoterapy with de-escalated radiotherapy (reducing total dose)
qq) Radio-chemoterapy with curative dose
rr) De-escalated radiotherapy (reducing total dose)
ss) Other (specify…)

21) What chemotherapy regimen do you think is the best combined with radiotherapy?

tt) 5-FU-MMC
uu) 5-FU-CDDP
vv) Capecitabine-MMC
ww) Capecitabine-CDDP
xx) Other (specify…)

22) In case of 5-FU/MMC o Cape/MMC chemotherapy regimen, how many MMC cycles do you normally administer to the patient?

yy) One (first RT week)
zz) Two (first and last RT week)
aaa) Other (specify…)

23) Which dose of MMC do you use?

bbb) In case of 1 MMC cycle= ….(mg/m^2)
ccc) In case of 2 MMC cycles= ….(mg/m^2)

24) Do you usually prescribe a pre-treatment DYPD (Dihydropyrimidine dehydrogenase) polymorphism screening in case of fluoropyrimidine-based chemotherapy regimens?

ddd) Yes
eee) No

25) How do you consider capecitabine in combination with MMC or CDDP concurrent to RT?

fff) Standard of care (for everyday clinical practice)
ggg) Investigational (only in clinical studies)
hhh) Potential option in case of patients' preference or in case of concerns for central venous catheter positioning
iii) Other (specify…)

26) How do you consider CDDP use in addition to 5FU/capecitabine, instead of MMC, combined with RT?

jjj) Equivalent to MMC
kkk) Inferior to MMC
lll) Not the standard of care, but optional in case of clinical contraindication to MMC use (i.e.: predicted hematological toxicity)
mmm) Other (specify…)

27) Do you think induction chemotherapy should be indicated before definitive chemo-radiotherapy for anal cancer?

mmm) Always
nnn) Never
ooo) Only in case of extensive disease (i.e.: lombo-aortic nodal involvement) or extensive pelvic nodal involvement
ppp) Other (specify…)

28) Do you think adjuvant chemotherapy should be indicated after definitive chemo-radiotherapy for anal cancer?

qqq) Ever
rrr) Never
sss) Only in case of high-risk disease (locally advanced tumors with positive nodes) or extensive pelvic nodal involvement
ttt) Other (specify…)

29) Which induction or adjuvant chemotherapy schedule do you use?

induction chemotherapy:…………..
adjuvant chemotherapy:………….

30) In your Center, for HIV patients under antiretroviral therapy, which approach do you use for radiochemotherapy?

uuu) Radio-chemotherapy with standard doses and schedules in any case
vvv) Radio-chemotherapy with standard doses and schedules in presence of regular CD4+ count
www) Radio-chemotherapy with standard doses and schedules in presence of regular CD4+ count and HIV RNA undetectable
xxx) Chemotherapy drugs are always reduced in this setting of patients
yyy) Alternative chemotherapy drugs (i.e.: CDDP instead of MMC)

31) Which is the right timing to evaluate response to chemoradiotherapy?

zzz) 8 weeks after the end of RT-CT
aaaa) 3 months after the end of RT-CT
bbbb) 6 months after the end of RT-CT
cccc) >6 months after the end of RT-CT
dddd) 26 weeks after the initiation of RT-CT

32) Which imaging examination do you think is indicated to assess response after RTCT in anal cancer patients? (multiple answers allowed)

eeee) Thorax and abdomen contrast-enhanced CT
ffff) Contrast-enhanced high resolution pelvic MRI including inguinal region
gggg) ^{18}FDG-PET CT
hhhh) Endo-anal ultrasound

33) When do you perform biopsy during evaluation of treatment response?

iiii) Always
jjjj) In case of suspicion for persistent disease or fibrotic residual disease
kkkk) In case of suspicion for persistent disease
llll) Evaluation in terms of tumor response’s clearance and type
mmmm) Never

34) In case of persistent or recurrent disease, do you consider salvage surgery as a curative treatment?

nnnn) Yes, always
oooo) Yes, in almost half of the patients
pppp) Never
qqqq) I discuss this therapeutic option during the multidisciplinary board
rrrr) Other (specify…)

35) Which approach do you use to manage recurrent disease?

ssss) Salvage surgery, if possible
tttt) Palliative reirradiation+ chemotherapy
uuuu) Definitive chemotherapy
vvvv) Preoperative reirradiation+ chemotherapy + surgery

36) In your center, in case of metastatic disease, which first line chemotherapy scheme is the standard of care?

zzzz) CDDP-5FU
aaaaa) CBDCA + Paclitaxel
bbbbb) (modified) Docetaxel + CDDP + 5-FU
ccccc) Other (specify…)

37) Which is the management for late toxicity and sequelae in long-survivors?

ddddd) I manage long-term follow-up personally
eeeee) I do not manage long-term follow-up personally
fffff) I rely on other colleagues (surgeon, medical oncologist)
ggggg) I work within the multidisciplinary tumor board

38) Which is the timing of follow-up for anal squamous cell carcinoma?

hhhhh) Every 3 months for 5 years
iiiii) Every 6 months for 5 years
jjjjj) Every 3 months within the first year and every 6 months for the following 4 years
kkkkk) Every 3 months within two years and every 6 months for the following 3 years
lllll) Other (specify…)
